# Supplementary material for: Comprehensive Annotation and Functional Exploration of MicroRNAs in Lettuce
Source: Front Plant Sci. 2021 Dec 24;12:781836. doi: 10.3389/fpls.2021.781836 (PMC8739914; doi:10.3389/fpls.2021.781836)
Supplement: Supplementary file 3 [file Data_Sheet_9.ZIP › GSM2230753.plot/Lsa-miRN1709_Lsat_1_v5_gn_8_157520.1_2224_TPlot.pdf]

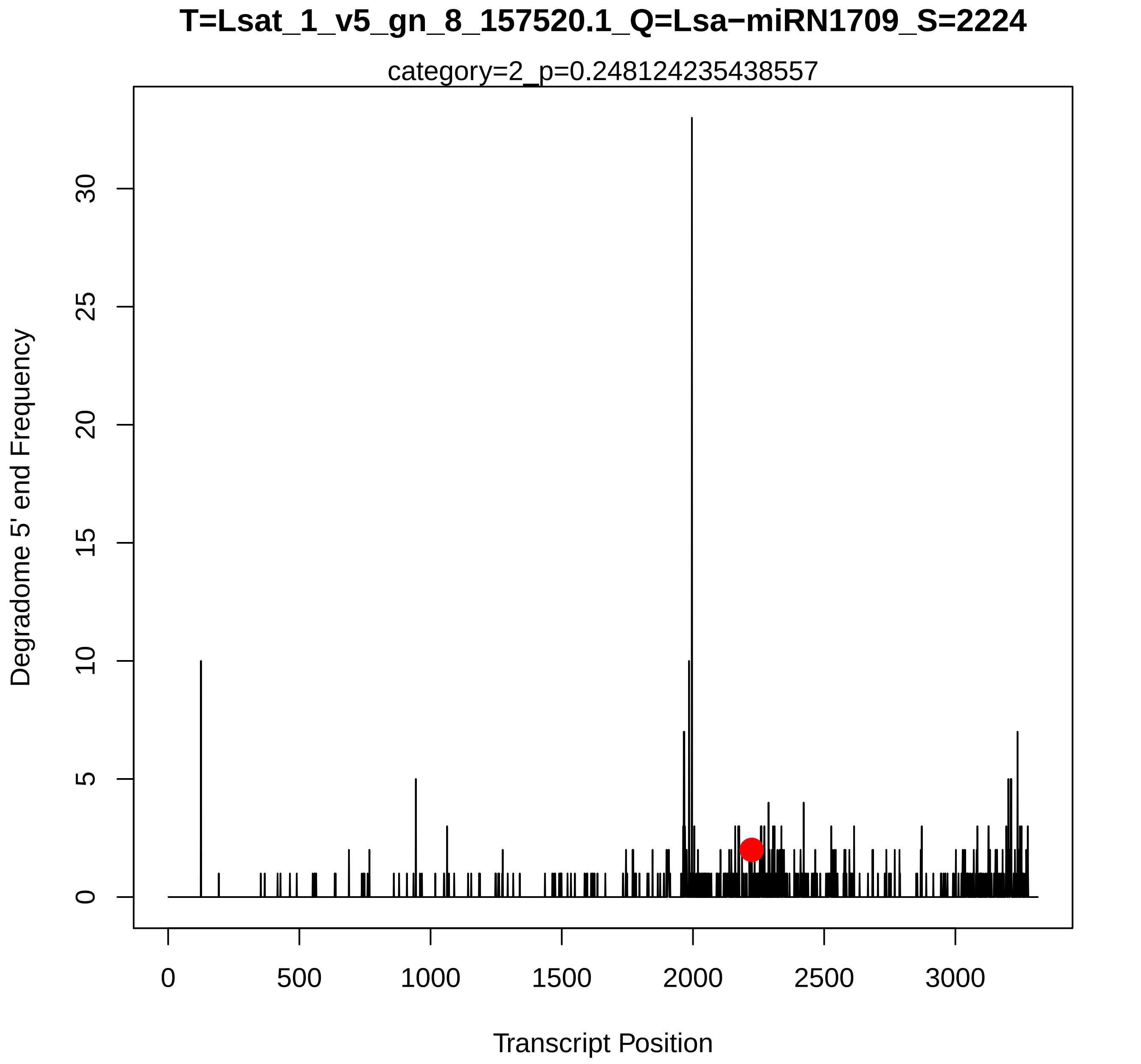

T=Lsat_1_v5_gn_8_157520.1_Q=Lsa−miRN1709_S=2224
category=2_p=0.248124235438557
Degradome 5' end Frequency
Transcript Position
0
5
10
15
20
25
30
0
500
1000
1500
2000
2500
3000
